# Aortic Mural Thrombus in a Critically Ill Patient: A Case Report

**DOI:** 10.51894/001c.144554

**Published:** 2025-09-30

**Authors:** Pallavi Makineni

**Affiliations:** 1 Department of Internal Medicine Detroit Medical Center-Sinai Grace Hospital

## 99

### INTRODUCTION

Aortic mural thrombus is typically associated with aneurysmal disease, dissection, or severe atherosclerosis but is an unusual cause of peripheral arterial embolization. Due to its rarity, the natural history and management of this condition are poorly understood, with limited case reports and small series available in the literature.

### CASE DESCRIPTION

An 86-year-old male with a history of dementia, hyperlipidemia, and hypertension presented with acute agonal breathing, hypotension, and unresponsiveness. The patient had semi-formed stools and a productive cough in the preceding days and was mostly nonverbal at baseline due to dementia. Initial examination revealed bilateral crackles on chest auscultation. The patient was intubated upon admission, and chest X-ray identified multifocal pneumonia. A CT scan of the abdomen and pelvis revealed a chronic aortic mural thrombus extending from the diaphragmatic hiatus to the aortic bifurcation, extensive atherosclerotic vascular calcifications, a small aortic aneurysm, and diffuse small bowel thickening suggestive of ischemia, despite patent mesenteric arteries. Blood and respiratory cultures confirmed Escherichia coli septicemia and Klebsiella pneumoniae infection. Complications included NSTEMI and atrial fibrillation with rapid ventricular response. Management included vasopressors, hydrocortisone, antibiotics, and heparin therapy. Despite these interventions, the patient suffered cardiac arrest and achieved return of spontaneous circulation after 8 minutes but ultimately passed away.

### CONCLUSION

This case highlights the complex interplay of factors leading to the patient’s decline, with aortic mural thrombus as a key contributor. In the context of atherosclerotic risk factors, NSTEMI, and atrial fibrillation, the thrombus likely caused mesenteric artery hypoperfusion, ischemic injury, and subsequent Escherichia coli translocation into the bloodstream, resulting in septicemia. The systemic inflammation and sepsis led to multifocal pneumonia, with E. coli and Klebsiella pneumoniae identified in respiratory cultures. These complications, along with NSTEMI and atrial fibrillation, caused worsening hemodynamic instability, culminating in cardiac arrest and death despite aggressive intervention. The case underscores the critical importance of early recognition and management to improve outcomes. Given the significant complications and high mortality associated with aortic mural thrombus, heightened suspicion is essential in at-risk patients. Further research is needed to establish standardized diagnostic and therapeutic strategies for this rare but life-threatening condition.

